# Dietary Erythrodiol Modifies Hepatic Transcriptome in Mice in a Sex and Dose-Dependent Way

**DOI:** 10.3390/ijms21197331

**Published:** 2020-10-04

**Authors:** Roubi Abuobeid, Luis Herrera-Marcos, María A. Navarro, Carmen Arnal, Roberto Martínez-Beamonte, Joaquín Surra, Jesús Osada

**Affiliations:** 1 Departamento de Bioquímica y Biología Molecular y Celular, Facultad de Veterinaria, Instituto de Investigación Sanitaria de Aragón-Universidad de Zaragoza, E-50013 Zaragoza, Spain; roubi.a.obeid@gmail.com (R.A.); l.vte.herrera@gmail.com (L.H.-M.); angelesn@unizar.es (M.A.N.); 2 Instituto Agroalimentario de Aragón, CITA-Universidad de Zaragoza, E-50013 Zaragoza, Spain; arnal@unizar.es (C.A.); romartin@unizar.es (R.M.-B.); jsurra@unizar.es (J.S.); 3 CIBER de Fisiopatología de la Obesidad y Nutrición, Instituto de Salud Carlos III, E-28029 Madrid, Spain; 4 Departamento de Patología Animal, Facultad de Veterinaria, Instituto de Investigación Sanitaria de Aragón-Universidad de Zaragoza, E-50013 Zaragoza, Spain; 5 Departamento de Producción Animal y Ciencia de los Alimentos, Escuela Politécnica de Huesca, Instituto de Investigación Sanitaria de Aragón-Universidad de Zaragoza, E-22071 Huesca, Spain

**Keywords:** erythrodiol, mice, liver, apolipoprotein E, olive oil, transcriptome

## Abstract

Erythrodiol is a terpenic compound found in a large number of plants. To test the hypotheses that its long-term administration may influence hepatic transcriptome and this could be influenced by the presence of APOA1-containing high-density lipoproteins (HDL), Western diets containing 0.01% of erythrodiol (10 mg/kg dose) were provided to *Apoe*- and *Apoa1*-deficient mice. Hepatic RNA-sequencing was carried out in male *Apoe*-deficient mice fed purified Western diets differing in the erythrodiol content. The administration of this compound significantly up- regulated 68 and down-regulated 124 genes at the level of 2-fold change. These genes belonged to detoxification processes, protein metabolism and nucleic acid related metabolites. Gene expression changes of 21 selected transcripts were verified by RT-qPCR. *Ccl19-ps2*, *Cyp2b10*, *Rbm14-rbm4*, *Sec61g*, *Tmem81*, *Prtn3*, *Amy2a5*, *Cyp2b9* and *Mup1* showed significant changes by erythrodiol administration. When *Cyp2b10*, *Dmbt1*, *Cyp2b13*, *Prtn3 and Cyp2b9* were analyzed in female *Apoe*-deficient mice, no change was observed. Likewise, no significant variation was observed in *Apoa1*- or in *Apoe-*deficient mice receiving doses ranging from 0.5 to 5 mg/kg erythrodiol. Our results give evidence that erythrodiol exerts a hepatic transcriptional role, but this is selective in terms of sex and requires a threshold dose. Furthermore, it requires an APOA1-containing HDL.

## 1. Introduction

A preeminent health-promoting traditional eating pattern commonly known as The Mediterranean diet (MedDiet) has occupied a wide framework of global research efforts for decades due to the very low-cardiovascular disease mortality rates regardless of lifestyle, poverty or any geographical differences between countries who have traditionally consumed as was evidenced by The Seven Countries study [1]. Further epidemiological studies revealed that this pattern was also associated with lower mortality rates and thus provided a healthy and extended life-span [2]. Recent evidence has proved that an intervention using this diet is able to reduce cardiovascular mortality [3]. A review of these aspects can be found in [4].

Virgin olive oil, directly extracted from fresh olive fruits, stands out as the major source of lipids in the MedDiet [5,6]. Pomace olive oil is a blend obtained from remainder of the olives, skin, and pits used organic solvents [7] and is also consumed in this geographical area. Both have similar fatty acid composition [5,8], but vary in phytosterols, waxes, tocopherols and triterpenes (uvaol, maslinic and erythrodiol) with higher content in pomace than in virgin olive oil [9]. Several recent studies have shown that continuous consumption of pomace olive oil protects against carcinogenic activities [10], hepatic steatosis [9], atherosclerosis [6], cardiovascular problems (CHD and stroke), inflammation and type 2 diabetes mellitus [5]. The latter was associated with positive antioxidant properties [11] or reduction in blood pressure [5].

**Figure 1:**
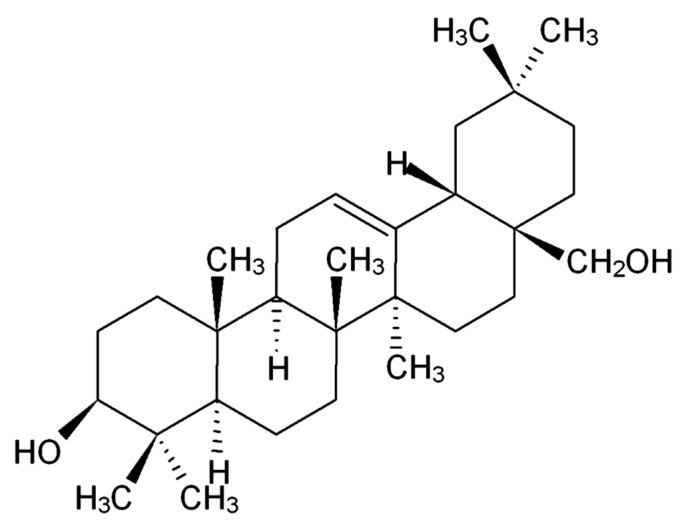
Erythrodiol chemical structure.

Erythrodiol (18b-olean-12-ene-3b,28diol) [7], a 30-carbon atom pentacyclic triterpene (Figure 1) is biosynthesized by a cascade of cyclizations and rearrangements from oxidosqualene [10,12,13]. The alcohol is present in virgin olive oil at a concentration of 75 mg/kg [9,13], with more presence in the unsaponifiable fraction of this oil [14] at a concentration of 500 mg/kg [9]. Erythrodiol is also detected in olive leaves [10,15], leading to approximately 60% of their triterpenic content [13]. This alcohol is widely distributed through other plant species including leaves of *Ficus mysorensis* [12], *Conyza canadensis* [16], *Celastrus kusanoi* stems [17], stem bark of *Erythrina indica* [18], birch bark trees [19] and leaves of *Maytenus ilicifolia* [20].

Several studies have shown favorable properties of erythrodiol including diverse endogenous anti oxidative activities [11], antiproliferative, proapoptotic actions against colon adenocarcinoma HT-29 cells [10], histolytic lymphoma (U937) cells [21], breast cancer [21], gastric cancer [16] and astrocytoma [22]. The triterpenoid is also related to several antioxidant, antithrombotic and vasorelaxant benefits against cardiovascular problems [23,24], combined with the ability to reduce cardiac hypertrophy and block profibrotic effects of angiotensin II [24]. Other biological activities include anti-inflammatory, immunomodulatory and anti-edematous properties by reducing neutrophil infiltration [8,14,25] and its ability to protect from neuroinflammation [25]. Erythrodiol was found to induce wound healing by increasing the production of actin filopodia, lamellipodia and stress fibers through activating Rho GTPases [19] and has provided antiplatelet properties when inhibiting ADP-induced activation [15].

Significant modifications on hepatic gene expression were found after long-term administration of pomace olive oil fraction-enriched diets with erythrodiol among other compounds [9]. Since the changes were not observed by its single administration, a new experiment was required to single out its action. In this regard, the long-term effect of erythrodiol needed to be addressed in order to understand and assess molecular functions and pharmacogenetic pathways of this triterpene.

## 2. Results

### 2.1. Somatometric Parameters

A long-term administration of a 10 mg/kg erythrodiol-supplemented Western diet was carried out in two mouse models of both sexes: *Apo1*- and *Apoe*-deficient mice. In the former group, erythrodiol administration for 12 weeks significantly increased body weight gain in males (4.2 ± 1.6 vs. 5.9 ± 1.4 g, *p* < 0.01 for control and erythrodiol, respectively). In contrast, in the second model, the administration of the triterpene for 4 weeks caused a decrease in body weight gain (4.5 ± 5.0 vs. 3.1 ± 3.3 g, *p* < 0.05 for control and erythrodiol, respectively) with a marked decrease in liver mass (1.6 ± 0.6 vs. 1.3 ± 0.5 g, *p* < 0.05 for control and erythrodiol, respectively). No effect was observed in females of both genetic models.

### 2.2. Histological Analyses

**Figure 2:**
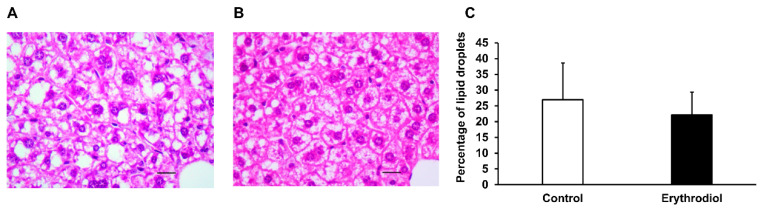
Hepatic histological analyses in male Apoe-deficient mice fed the different diets. Representative liver micrographs at 400× magnification from consuming a Western diet (**A**) and consuming a 10 mg/kg erythrodiol-containing Western diet (**B**). Liver sections (4 μm) from each mouse were stained with hematoxylin and eosin and blind evaluated. Bars denote 20 μm. Morphometric changes of hepatic fat surface in mice consuming the different diets (**C**) where data are means ± SD for each group (*n*= 14 and *n* = 15, respectively for control and erythrodiol). Statistical analyses were done according to Mann–Whitney’s U-test.

Mice lacking APOE are models of spontaneous hepatic steatosis as shown in Figure 2A. To explore the influence of erythrodiol administration on this parameter, histological analyses of livers were carried out. Male *Apoe*-deficient mice consuming a 10 mg/kg erythrodiol-enriched Western diet for 12 weeks showed decreased lipid droplets (Figure 2B). When the fat areas were quantified, the group receiving erythrodiol exhibited a non-significant (*p* < 0.06) trend to a lesser accumulation than control group, as shown in Figure 2C.

### 2.3. Hepatic Gene Expression of Apoe-Deficient Male Mice Fed for 12 Weeks on a 10 mg/kg Erythrodiol-Containing Western Diet

To determine the impact of erythrodiol intake on hepatic transcriptome, seven RNA pools from fifteen animals receiving the above diet and another seven from fourteen mice receiving the Western control diet were sequenced using next generation sequencing. From each library, clean reads sequences (46,765 × 10^3^ ± 7189 × 10^3^), filtered from contaminants, adaptors, low quality regions, and reads with unknown bases, were mapped onto reference genome, followed by gene prediction. In both groups, the mapping ratio was 74% for a transcript number of 31,369 ± 3051. Splicing patterns contributed to a variety of differentially splicing genes with a total of 14,920 novel transcripts, 13,439 coding and 1481 non-coding transcripts. Coding genes showed 11,605 previously unknown splicing events for known genes and 1834 coding transcripts previously unknown. Globally, the erythrodiol administration did not significantly modify single nucleotide polymorphisms. In this regard, transversions A–G were 3172 ± 1019 vs. 3835 ± 155 for control and erythrodiol groups, respectively, and those corresponding to C–T were 3113 ± 1024 vs. 3787 ± 133. No significant changes were observed for transversions either (A–C, 609 ± 209 vs. 743 ± 47; A-T 796 ± 335 vs. 988 ± 61; C–G, 576 ± 217 vs. 726 ± 23 and G-T, 578 ± 200 vs. 708 ± 28 for control and erythrodiol groups, respectively).

When alternative splicing events were screened, erythrodiol administration had no significant effect. Skipped exons were 6432 ± 1526 vs. 7178 ± 344 for control and erythrodiol groups; alternative 5′ splicing sites, 1568 ± 368 vs. 1789 ± 42; alternative 3′ splicing sites: 2181 ± 522 vs. 2503 ± 62; mutually exclusive exons, 696 ± 102 vs. 732 ± 48 and retained introns, 1504 ± 265 vs. 1688 ± 20 in control and erythrodiol groups, respectively.

**Figure 3:**
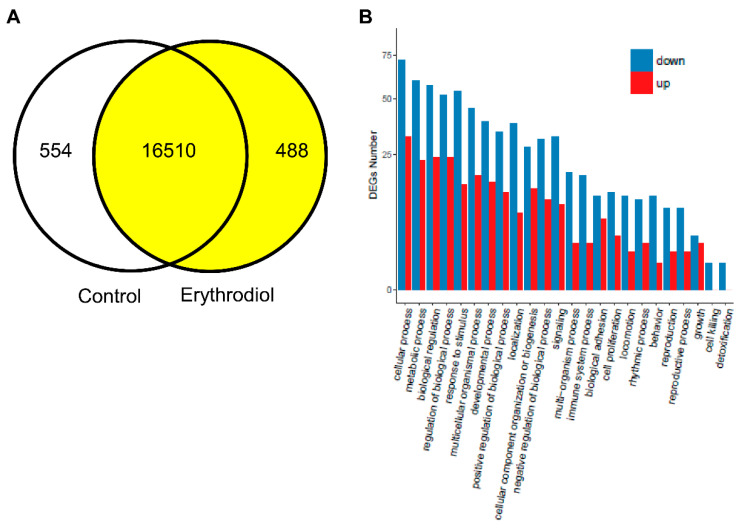
Differentially expressed genes. (**A**), Venn diagram analysis. Control expressed 17,064 genes, while erythrodiol expressed 16,998 genes. Functional enrichment analysis of differentially expressed genes. (**B**), Gene ontology (GO) classification of biological processes of liver transcriptome by erythrodiol administration. X axis represents GO term. Y axis represents the amount of up/down-regulated genes. DEGs, differentially expressed genes.

**Figure 4:**
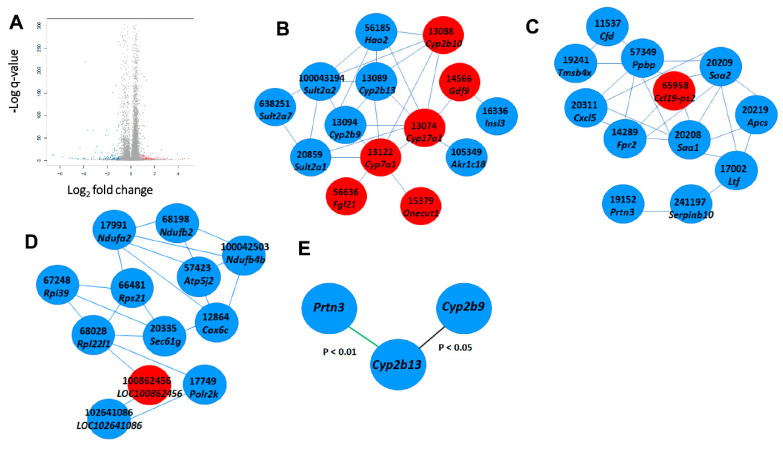
Significant expression changes and major networks involved. (**A**), Volcano plot representing control vs. erythrodiol differentially expressed genes; 68 up regulated genes and 124 down-regulated with false discovery rate *q* < 0.001; 16.375 no change with 2 fold change. (**B**), network of genes involved in detoxification, (**C**), network of genes involved in protein metabolism and (**D**), network associated with DNA. Red color denotes up-regulation while blue corresponds to down-regulation. (**E**), significant association of genes expressions assayed with RT-qPCR.

Differentially expressed genes, shown in Figure 3A, were 554 in the control and 488 in the erythrodiol group. According to their gene ontology classification, Figure 3B, all kinds of biological processes were involved, being cellular processes the category that included the highest number of genes. Using more stringent criteria of 2-fold change and false discovery rate of *p* < 0.001, 68 up- regulated genes and 124 down-regulated as reflected in the volcano plot of Figure 4A. As reflected in Figure 4B–D, these genes were sorted into three main categories: detoxification processes, protein metabolism and nucleic acid related compounds. An example of genes modified at the level 2.9-fold change (log2 fold change 1.5 or −1.5) is reflected in Table 1 and Table 2. Twenty-nine transcripts were up-regulated (Table 1) and sixty-three were down-regulated according to this criterion (Table 2).

To confirm the RNAseq data carried out on seven hepatic RNA pools of each group, 21 transcripts were randomly chosen from Table 1 and Table 2 to design their RT-qPCR assays. The latter were carried out on individual hepatic RNA samples of each mouse. Selected transcripts were: *H4c17*, *LOC100862456*, *Ccl19-ps2*, *Ctrb1*, *Cyp2b10*, *Zfp969*, *Zfp965*, *Ttn*, *Rbm14-rbm4*, *Sec61g*, *Rbm24*, *Tmem81*, *Rnase2a*, *Sult2a2*, *Ndufb4b*, *Dmbt1*, *Cyp2b13*, *Prtn3*, *Amy2a5*, *Cyp2b9* and *Mup1*. The expression of these transcripts normalized to the average of *Ppib* and *Tbp* reference genes is depicted in Table 3. Only 9 (*Ccl19-ps2*, *Cyp2b10*, *Rbm14-rbm4*, *Sec61g*, *Tmem81*, *Prtn3*, *Amy2a5*, *Cyp2b9* and *Mup1*) out of 21 showed significant changes by the administration of erythrodiol. Association analyses of individual values obtained by RT-qPCR of these genes (Figure 4E) revealed a significant association between *Cyp2b13* and *Cyp2b9* and between *Cyp2b13* and *Prtn3*, suggesting a certain co-regulation or overlapping in biological activities.

**Figure 5:**
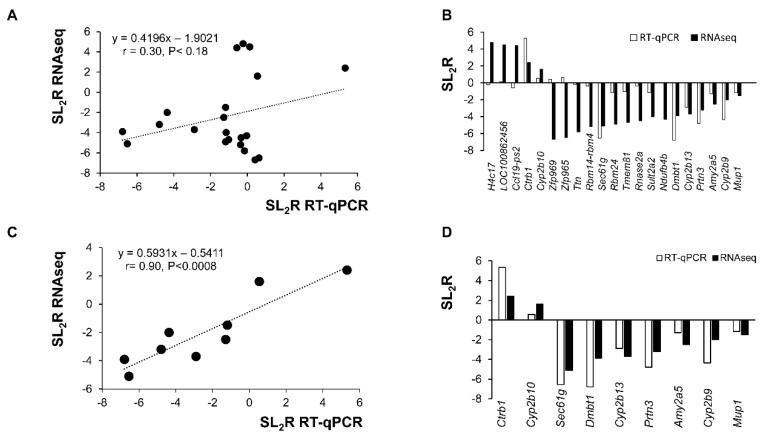
Concordance between used methods of RNA analysis. (**A**) Correlation analysis of 21 selected genes between RNAseq and RT-qPCR normalized to the invariant *Pipb* and *Tbp* genes. The mean values obtained for signal log_2_ ratio (SL_2_R) from individual analyses (Table 3) were plotted against the RNAseq which used partially pooled samples (Table 1 and Table 2). Poor agreement between the procedures was seen (*r* = 0.3, *p* < 0.18). (**B**) Changes in values of SL_2_R expression of both methods for the 21 selected genes. (**C**) SL_2_R correlation analysis between between RNAseq and RT-qPCR results normalized to the invariant *Pipb* and *Tbp* of 9 filtered genes after excluding those without counts in more than 60% of samples. Good agreement between the procedures was observed (*r* = 0.8, *p* < 0.0008). (**D**) Changes in values of SL_2_R expression of both methods for the 9 after removing those with low counts.

Using the log_2_ ratio of fold changes obtained by RNAseq and RT-qPCR for the twenty-one selected transcripts, a correlation analysis was carried out. As shown in Figure 5A, a non-significant low correlation coefficient of 0.3 was obtained. Indeed, as shown in Figure 5B, there were important discrepancies between both methods. To explore the reason of such lack of agreement, both methods were critically revised. When transcripts of RNAseq showing either zero counts or without counts in more than 60% of samples were excluded, a significant agreement (*r* = 0.9, *p* < 0.0008) between both methods was observed (Figure 5C) and all samples were properly categorized (Figure 5D). Only 9 out the 21 chosen genes tested by RT-qPCR show good correlation with the RNA seq data.

### 2.4. Hepatic Gene Expression in the Livers of Female Apoe-Deficient Mice Fed on a 10 mg/kg Erythrodiol-Containing Western Diet for 12 Weeks

To explore, a sex-related response, five transcripts (*Cyp2b10*, *Dmbt1*, *Cyp2b13*, *Prtn3 and Cyp2b9*) showing high expression changes in male *Apoe*-deficient mice were used as subrogated markers of erythrodiol administration and quantified their expression in the female livers. Results showed no significant changes (Table 4). This finding points out to a sex-specific hepatic gene expression in response to erythrodiol.

### 2.5. Influence of Erythrodiol Dose on Selected Hepatic Gene Expressions in Male Apoe-Deficient Mice Fed on Erythrodiol-Containing Western Diets for 12 Weeks

A putative dose–response relationship was examined in males for the five selected genes, *Cyp2b10*, *Dmbt1*, *Amy2a5*, *Prtn3* and *Cyp2b9.* Several doses ranging from 0.5 to 5 mg/kg were tested in male *Apoe*-deficient mice. Interestingly, none showed a significant change in gene expression (Table 5). Genes such as *Cyp2b10* at 0.5 mg/kg and *Dmbt1* at 1 mg/kg showed no normal distributions with individual highly responders that skew the standard deviation and force the statistical analysis to a non-parametric approach. Despite the significant odd increases in *Cyp2b9* at 0.5 mg/kg and *Prtn3* at 1 mg/kg, no general trend of more pronounced changes was observed when a higher dose was used. These results suggest that 10 mg/kg is the lower effective dose contributing to inducing hepatic gene expression changes.

### 2.6. Influence of Apoa1-Deficiency on Selected Hepatic Gene Expressions on Mice Consuming the 10 mg/kg Erythrodiol-Containing Western Diet for Four Weeks

Absence of APOA1 is a genetic model of HDL deficiency. In mice lacking this protein from both sexes, the impact of an erythrodiol-containing Western diet on hepatic gene regulation was assessed by measuring the expressions of *Cyp2b10*, *Dmbt1*, *Cyp2b13*, *Prtn3 and Cyp2b9* as subrogate genes. Results showed no significant change in any of the genes in either sex following erythrodiol administation (Table 6). These results may imply that APOA1-containing HDL may participate in delivering erythrodiol to the liver.

## 3. Discussion

The present nutrigenomic approach was carried out to determine the effect of erythrodiol on hepatic transcriptome in male *Apoe*-deficient mice as a hepatic steatosis-prone model. Using RNAseq, erythrodiol administration did not modify single nucleotide polymorphisms, nor created errors in transcription, nor influenced global alternative splicing events. Results indicate that this compound mainly modified hepatic expression of clusters of genes involved in xenobiotics, protein and nucleic acid metabolisms. These findings were accompanied by a trend to decrease accumulation of lipids in cytoplasmic lipid droplets and decreased hepatic mass. A comparison between RNAseq and RT-qPCR revealed that due to their different methodological approaches, special care should be applied in order to compare their outcomes. Nine randomly selected genes *(Ccl19-ps2*, *Cyp2b10*, *Rbm14-rbm4*, *Sec61g*, *Tmem81*, *Prtn3*, *Amy2a5*, *Cyp2b9* and *Mup1)* showing good agreement between both methods were significantly modified in males by the administration of erythrodiol. An association of expressions among *Cyp2b13*, *Cyp2b9* and *Prtn3* was observed. When these gene expressions together with that of *Cyp2b10* and *Dmbt1* were tested in female mice receiving erythrodiol, a different sex-response was observed. Used to explore the minimal required dose, they evidenced a minimum 10 mg/kg, to observe male responses. Used as markers of erythrodiol delivery to the liver in absence of APOA1-HDL, no influence was observed in this setting. Overall, dietary erythrodiol administration is safe and induces hepatic gene changes that are sex-specific and dose-dependent and APOA1- containing HDL may participate in its delivery to the liver.

Due to the fact that the high-throughput sequencing technology for transcriptomic purposes provides huge amounts of data about differentially expressed genes and additional analyses including polymorphisms, alternative spliced variants, low-expressed genes, and novel transcripts, has been proposed as an attractive choice, superseding quantitative transcript profiling by microarray [26]. Using this approach, we have proved that erythrodiol administration induced differentially expressed genes without modifying single nucleotide polymorphisms, creating errors in transcription or influencing global alternative splicing events. This fact, the lack of dead mice receiving this agent at 10 mg/kg for 12 weeks, and the normal hepatic morphology indicate that erythrodiol administration is safe for males and females.

In our technical approach for RNA seq, a strategy of pooling was adopted. This may raise two drawbacks, bias and loss of biological variability, but it also has advantages in terms of cost and complexity of analysis [26,27]. Undoubtedly, this approach requires confirmation by an independent procedure, and RT-qPCR was selected and applied to 21-randomly chosen genes that were analyzed using individual samples. The initial correlation between RNAseq and RT-qPCR was rather poor (Figure 5A). A profound analysis of both methods considering primer design used in RT-qPCR that corresponded to most read exons, and establishing an unambiguous limit of detection in RNAseq showed a robust agreement between both procedures (*r* = 0.9, *p* < 0.0008) (Figure 5C). Thereby, pooling assays are a reliable screening approach that saves samples, is more economic and straightforward as it was for microarrays [9,28,29] and in this particular case it also facilitates the finding of targets of erythrodiol. RNAseq provides an unsurpassed overview of genome activity. Although preliminary, this finding also suggests that bioinformatic tools for analyzing RNAseq data should be refined to reinforce their specificity in displaying quantified transcripts and taking into consideration their limits of detection. One potential limitation of our approach is the search in the range of the highest changes where some control samples did not show any expression and did contribute to an increased erythrodiol/control ratio. For that reason, only 9 out 21 chosen genes show good correlation. Furthermore, it should also be taken into consideration that depending on the chosen primers, different transcripts are analyzed [30]. These caveats warrant more research in the future.

A cluster of genes involved in xenobiotics metabolism has been influenced by erythrodiol administration. In this regard, one gene with induced expression was *Cyp2b10*. It belongs to cytochrome P450 components of phase I response involved in NADPH-dependent electron transport. It oxidizes steroids, fatty acids, and xenobiotics, leading to the detoxification of approximately 10% of drugs [31]. Erythrodiol as an alcohol was able to induce it, as did ethanol [32]. However, other members of phase I response, *Cyp2b9* and *Cyp2b13*, were found decreased by erythrodiol administration. This represents a unique pattern differing from the response to oleanolic acid-diet that also induced *Cyp2b9* expression [28] and that of maslinic acid administration that induced the triad (*Cyp2b9*, *Cyp2b10* and *Cyp2b13*) [29] or the lineal triterpene, squalene (*Cyp2b10* and *Cyp2c55*) [33]. These facts clearly indicate different responses depending on the administered triterpene. This particular hepatic response to erythrodiol may escape of the consequences observed when these three *Cyp* genes were deleted, namely fatty liver disease progression [34]. Indeed, our results do not support an increased lipid droplet accumulation in the liver.

Regarding protein metabolism, particularly interesting are the findings of *Prtn3* changes and their association with those of *Cyp2b9* and *Cyp2b13* (Figure 4E). *Prtn3* encodes for a proteinase 3 with proteolytic activities and reactive oxygen species responses. *Ptrn3* deficiency is strongly correlated with fewer incidences of liver steatosis and adipose tissue inflammation and thus reduced risk of NAFLD and obesity-related steatosis [35]. High PRTN3 levels are also correlated with poor survival rates in pancreatic cancer [36]. In this regard, *Ptrn3* suppression by erythrodiol may be a potent hepatic therapy against steatosis. *Mup1* is a member of the lipocalin family that regulates metabolic homeostasis by controlling the expression of gluconeogenic and lipogenic genes in the liver. Its down-regulation has been shown to induce hyperglycemia, impaired insulin secretion, glucose intolerance and hyperlipidemia [37,38]. Accordingly, erythrodiol could carry deleterious effects when repressing MUP1 gene. On the other hand, SEC61g over-expression is considered a parameter of bad glioblastoma prognosis [39]. TMEM81 is overexpressed in hepatocellular carcinoma [40]. In addition, a frameshift deletion mutation of RBM14-RBM4 chimera was screened in liver cancer, considering this mutation a putative marker for hepatic neoplasia [41]. The decreased expressions of these three genes by erythrodiol might contribute to explaining its antineoplastic properties [22].

When comparing gene expression patterns of our study with those observed using olive oil components, *Dmbt1*, an extracellular receptor, showed reduced expression in animals consuming a pomace olive diet [9]. This pattern was reproduced by erythrodiol but not by other terpenic compounds. Thus, the gene could be a unique marker for erythrodiol intake. Its significance on liver damage needs to be explored [42].

The sex-differences noted in gene expression changes observed by erythrodiol administration are particularly striking. Our results again reinforce the previously noted differences between sexes in the liver [43], particularly when a Western diet was administered [30]. Indeed, *Cyp2b*9 gene expressions have been particularly sensitive to sex differences [34,44,45], β-estradiol [46] and prolactin [47]. Thus, hepatic drugs should be specially tested for females. A threshold dose was also required to elicit male hepatic changes in gene expression confirming a dose-dependent pattern of other erythrodiol–related actions [10,25], and a potential cytotoxic effect at high doses [48]. APOA1 is the most abundant protein constituent of HDL produced by the liver and the intestine [49]. To test the hypothesis whether this type of HDL could be involved in delivering erythrodiol to the liver, mice lacking *Apoa1* gene were used as models of HDL absence. As suspected, no notable changes were observed for tested genes. The same results were obtained with oleanolic acid administration [28], which is consistent with the fact that both triterpenes may be vehicled by APOA1-containing HDL on their route to the liver. Overall, the hepatic gene expression profile induced by erythrodiol is a multistep, complicated process of integrated factors, particularly dose and sex. To translate these results into humans, its bioavailability has to be proved, something that has only been observed in rats [50]. Based on the present work and the different metabolic rates of mice and humans a 1 mg/kg dose should be explored in the latter. Consuming a daily 50 mL of extra-virgin olive oil, a human would be exposed to a 53 μg/kg erythrodiol dose, but using the same amount of pomace olive oil [51] the exposure would be 500 μg/kg, close to the predicted active dose in humans. A potential sex-differential response should be tested in our species.

## 4. Materials and Methods

### 4.1. Animal Models

The experimental animals used were two-month-old, homozygous *Apoe*-deficient mice of C57BL/6J genetic background, obtained from Charles River Laboratories (Barcelona, Spain) and *Apoa1*-deficient mice of C57BL/6J genetic background, generously provided by Dr. Nobuyo Maeda from the University of North Carolina at Chapel Hill. Both were bred at the *Centro de Investigación Biomédica de Aragón*. Blood samples obtained from the facial vein 4h after a fasting period were used to establish experimental groups with similar baseline plasma cholesterol. This was used as a quick additional quality control of mouse identification. In this sense, *Apoe*-deficient mice on C57BL/6J background are hypercholesterolemic and their plasma levels should be higher than 5 ± 1 mmol/L [52] compared to 2.9 ± 0.5 mmol/L for wild-type. Any mouse not showing these values was genotyped and if their identification was correct were excluded due to hepatic dysfuntionality. In the case of *Apoa1*-deficient mice and due to their hypocholesterolemia [53], they should show values of 0.8 ± 0.4 mmol/L [54]. As in the case of *Apoe-*deficient mice, any discrepant mouse is genotyped as described [55]. The lack of *Apoe* and *Apoa1* hepatic expressions in their corresponding mice was also verified by analyzing their presence or absence by RT-PCR (Figure S1).

Mice were housed in sterile filter-top cages in rooms supplied with a monitored 12-h light/12-h dark cycle and had ad libitum access to food and water. The experiments were carried out in accordance with the EU Directive 2010/63 on the protection of animals used for scientific purposes and the study protocol was approved by the Ethics Committee for Animal Research of the University of Zaragoza (PI43/15, October 9th 2015 and PI35/18, October 4th 2018).

### 4.2. Experimental Designs

#### 4.2.1. Effect of Dietary 10 mg/kg Erythrodiol in a Western Diet on *Apoe*- and *Apoa1*-Deficient Mice

Four study groups were established: female (12) and male control (*n* = 14) groups received a purified Western diet containing 0.15% cholesterol and 20% refined palm oil (Gustav Heess, S.L., Barcelona, Spain), and the other two groups, female (13) and male (*n* = 15), were fed with the same diet containing 0.01% erythrodiol (Extrasynthese, Genay, France). Assuming a daily intake of 3 g for each mouse, this is equivalent to a dose of 10 mg/kg mouse. This dose was chosen based on that previously used of oleanolic acid [28] that did not modify body weight and elicited hepatic gene expressions. Fresh diets were prepared weekly, kept under N_2_ atmosphere at −20 °C and replaced daily. The animals were fed the experimental diets for 12 weeks and both were well tolerated.

A similar design was used for *Apoa1*-deficient mice; the groups were female (9) and male (14) controls, and female (9) and male (15) erythrodiol groups. In this case, the intervention lasted for 4 weeks.

#### 4.2.2. Effect of Different Doses of Erythrodiol in Western Diets on Male *Apoe*-Deficient Mice

Four groups were established. The control group (17) received the Western diet and three groups receiving the same diet formulated to receive doses of 0.5 (*n* = 16), 1 (*n* = 17) and 5 mg/kg erythrodiol (*n* = 17). As mentioned above, and once corrected by mouse metabolic rate, these doses would represent the amount of erythrodiol received by humans consuming extra-virgin olive oil or pomace olive oil. The animals were fed the experimental diets for 12 weeks.

### 4.3. Somatometric Analyses

During the experiment, body weight and survival rate were monitored. At the end of the experiment, following a four-hour fast, mice were euthanized by CO_2_ inhalation, and the livers obtained and weighed. An aliquot was stored in neutral formaldehyde and the remaining organ frozen in liquid nitrogen.

### 4.4. Liver Histology Analyses

Sections (4 μm) of the livers stored in neutral formaldehyde were stained with hematoxylin and eosin and observed using a Nikon microscope. Hepatic fat content was evaluated by quantifying the area of lipid droplets in each section and expressed as percentage of total liver section [56].

### 4.5. RNA Isolation

Total RNA of each liver was isolated using Tri Reagent from Ambion^®^ (Life Technologies, Carlsbad, CA, USA) following the manufacturer’s instructions. DNA contaminants were removed by TURBO DNAse treatment of 5 μg of total RNA using the DNA removal kit from Invitrogen (Cat.No:AM1907, Carlsbad, CA, USA). RNA was quantified by absorbance at A_260_/_280_ using Nanodrop Spectrophotometer and the ratio was greater than 1.75. The integrity of the 28S and 18S ribosomal RNAs was verified by 1% agarose gel electrophoresis followed by ethidium bromide staining and the 28S/18S ratio was greater than 2.

### 4.6. RNAseq and Data Analyses

For RNA sequencing, 6 pools of control mice were prepared using equal amounts of hepatic total RNA of two mice and in the seventh the total RNA from three mice was used. Another 7 pools were prepared for erythrodiol-treated mice combining total RNA from two or three mice per pool. The resulting 14 samples were sent to the Beijing Genomics Institute (BGI Genomics, Shenzhen, China) service. Their total RNA quality was tested using an Agilent 2100 Bioanalyzer (Agilent RNA 6000 nano kit, Santa Clara, CA, USA), then library construction was initiated by purifying the poly-A containing mRNA molecules using oligo-dT attached to magnetic beads. The mRNA was fragmented, copied into cDNA, linked to an adapter, purified and amplified by PCR. PCR yield was quantified by Qubit, and pooled samples together to make a single strand DNA circle (ssDNA circle), which gave the final library. DNA nanoballs (DNBs) were generated with the ssDNA circle by rolling circle replication (RCR) to enlarge the fluorescent signals at the sequencing process, the DNBs were loaded into the patterned nanoarrays and pair-end reads of 100 bp were read through the BGISEQ-500 platform. Sequencing reads which contained low-quality, adaptor-polluted and high content of unknown base reads were removed before downstream analyses. After read filtering, genome mapping of clean reads to reference genome was performed using HISAT (Hierarchical Indexing for Spliced Alignment of Transcripts), generating a Bioinformatics flow of about 4.73 Gb per sample with an average genome mapping rate of 92.76%. After genome mapping, StringTie was used to reconstruct transcripts [57], with genome annotation information, novel transcripts were identified by using Cuffcompare (a tool of Cufflinks) [58] and the coding ability of those new transcripts was predicted using Coding Potential Calculator [59]. In total, 14,920 novel transcripts were identified. GATK (Broad Institute, Inc, Boston, MA, USA) was then used to call SNP and INDEL variants for each sample. RMATS [60] was used to detect differentially splicing genes between samples. After novel transcript detection, novel coding transcripts were merged with reference transcripts to obtain a complete reference, then clean reads were mapped to it using Bowtie2 [61]. Then, gene expression level for each sample were calculated with RSEM [62]. The complete datasets were deposited in the GEO database (Accession number GSE155163).

### 4.7. Quantification of mRNA

To verify the most striking observed changes by the administration of erythrodiol using RNAseq, represented by signal log_2_ ratio > or <1.5 and false discovery rate <0.001 for up-regulated and down-regulated, respectively, 21 genes fulfilling these criteria were chosen. Their gene structure was analyzed using Ensembl Genome Browser and primers representative of the main hepatic transcripts according to Mouse Genome Informatics were prepared. The reverse transcriptase quantitative PCR (RT-qPCR) assays of these transcripts were optimized in terms of primer and input cDNA concentrations to obtain similar efficiencies and analyzed on individual samples. Basically, equal amounts of DNA-free RNA (500 ng) from each liver were reverse transcribed into cDNA using PrimeScript RT reagent Kit (Cat. No: RR037A, Takara, Kutsatsu, Shiga, Japan). The used primers were designed using NCBI and Primer 3 software [63] and checked by BLAST (NCBI) and KEGG to verify gene specificity and coverage of all variants for a specific gene. Table S1 depicts their characteristics. Quantitative real time was carried out according to manufacturer’s instructions (SYBR Green PCR Master Mix, Applied Biosystems, Foster City, CA, USA) on a Step One Real Time PCR System (Applied Biosystem). The relative amount of mRNA was calculated using the comparative 2^−ΔΔCq^ method and normalized to the reference *Ppib* and *Tbp* expressions and reported as signal log_2_ ratio of erythrodiol/control.

### 4.8. Quality Control and Statistics

PCR duplicates for samples were carried out and their coefficient of variation obtained. Samples displaying values higher than 3% were discarded and assayed again. High and low extreme data were also confirmed. Statistical analyses were performed using GraphPad Prism (GraphPad Software, San Diego, CA, USA). Data were checked for normal distribution by Shapiro–Wilk test and homogeneity of variance by Bartlett F-test. When any of these failed, results were analyzed by Mann–Whitney U test. Differences between both groups were considered significant when *p* < 0.05. Correlation between gene expressions was analyzed using Spearman correlation coefficient.

## 5. Conclusions

Through transcriptomic profiling and selecting a procedure previously validated by our group, erythrodiol has been proven to act as a transcriptional modulator of hepatic gene expression dependent on sex. At 10 mg/kg, erythrodiol modulates expression of hepatic genes involved in detoxification and tumor processes, and shows a trend of decreasing the percentage of area occupied by lipid droplets. In this aspect, erythrodiol could be a potential candidate to halt the evolution of fatty liver into hepatocarcinoma.

## Figures and Tables

**Table 1:** Hepatic transcripts differentially up-regulated by the administration of erythrodiol at the level of signal log_2_ ratio (SL_2_R) > 1.5 and false discovery rate < 0.001 in male Apoe-deficient mice according to RNAseq.

Biological Process	GenBank	Name	Gene Symbol	SL_2_R	*p*-Value
Nucleosome assembly	NM_001195421.1	H4 clustered histone 17	*H4c17/Hist1h4m*	4.8	0.0001
Transcription factor	XM_006537451.3	DNA-directed RNA polymerases I, II, and III subunit RPABC4	LOC100862456	4.5	0.001
Immune response	XM_006536385.3	Chemokine (C-C motif) ligand 19, pseudogene	*Ccl19-ps2*	4.4	0.001
Ion transport	NM_011644.3	Xndc1-transient receptor potential cation channel, subfamily C, member 2	*Xntrpc*	4.0	0.000
Cell differentiation, signaling activity	NM_183282.2	Actin-like 9	*Actl9*	3.1	0.0003
Proteolysis	NM_025350.4	Pancreatic carboxypeptidase A1	*Cpa1*	2.9	0.001
NA	XM_017321851.2	Extensin-like isoform X2	*Gm40365*	2.7	0.000
Chromosomal stability	NM_178212.3	H2A clustered histone 19	*H2ac19/Hist2h2aa2*	2.6	0.000
Aminopeptidase activity	NM_029008.1	Laeverin	*Lvrn*	2.5	0.001
G protein-coupled receptor signaling	NM_146337.1	Olfactory receptor 1396	*Olfr1396*	2.5	0.001
Proteolysis	NM_025583.2	Chymotrypsinogen B1	*Ctrb1*	2.4	0.000
NA	XM_017313070.1	Predicted gene/ coiled-coil domain containing 168	*Gm8251/Ccdc168*	2.2	0.000
Ion transmembrane transport	NM_001099298.3	Sodium channel, voltage-gated, type II, alpha	*Scn2a*	2.2	0.000
Cell adhesion	NM_001033364.3	Cadherin-related family member 2	*Cdhr2*	2.0	0.000
Short-term neuronal synaptic plasticity	NM_172737.4	Shisa family member7	*Shisa7*	2.0	0.0003
Regulation of immune response	NM_178786.4	Selection and upkeep of intraepithelial T cells 4	*Skint4*	1.9	0.001
Chromatin organization	NM_139218.1	Developmental pluripotency-associated 3	*Dppa3*	2.0	0.001
Transcription factor	NM_001029933.3	Zinc finger protein 114	*Zfp114*	1.9	0.001
Ubiquitin-protein transferase activity	NM_027708.1	F-box protein 24	*Fbxo24*	1.8	0.0002
Protein glycosylation and carbohydrate metabolism	NM_008051.6	Fucosyltransferase 1	*Fut1*	1.8	0.000
G protein-coupled receptor	NM_001011852.2	Olfactory receptor 1029	*Olfr1029*	1.7	0.001
NA	NA	Predicted gene, 40600	*Gm40600*	1.7	0.0001
Cell adhesion	NM_178685.5	Protocadherin 20	*Pcdh20*	1.7	0.000
Metal ion binding	NM_001220499.3	Ring finger 223	*Rnf223*	1.7	0.001
NA	NM_029608.1	Family with sequence similarity 209	*Fam209*	1.6	0.0003
P450 pathways	NM_009999.4	Cytochrome P450, family 2, subfamily b, polypeptide 10	*Cyp2b10*	1.6	0.000
NA	NM_027511.1	Histidine rich carboxyl terminus 1	*Hrct1*	1.6	0.001
Cell adhesion	NM_033585.2	Protocadherin gamma subfamily A, 2	*Pcdhga2*	1.5	0.000
Retinoic acid binding	NM_029958.1	Lipocalin 12	*Lcn12*	1.5	0.0001

NA, not available.

**Table 2:** Hepatic transcripts differentially down-regulated by the administration of erythrodiol at the level of signal log_2_ ratio < 1.5 and false discovery rate < 0.001 in male *Apoe*-deficient mice according to RNAseq.

Biological Process	GenBank	Name	Gene Symbol	SL_2_R	*p*-Value
Transcription factor	XM_017319408.2	Zinc finger protein 969	*Zfp969*	−6.7	0.0000
Transcription factor	NM_001242944.1	Zinc finger protein 965	*Zfp965*	−6.5	0.0000
Muscle structure	NM_011652.3	Titin	*Ttn*	−5.8	0.0000
Transcription factor	NM_001290127.1	RNA binding motif protein 14(Rbma4) and RNA binding motif protein 4 (Rbm4)	*Rbm14-rbm4*	−5.2	0.0000
Protein transmembrane transporter activity	NM_011343.3	Translocase Sec61 gamma subunit	*Sec61g*	−5.1	0.0000
Cell differentiation	NM_001081425	RNA binding motif protein 24	*Rbm24*	−4.9	0.0001
NA	NM_029025.3	Transmembrane protein 81	*Tmem81*	−4.7	0.0002
NA	NA	Nuclear body protein SP140-like	LOC105247075	−4.5	0.001
Metal ion binding, nucleic acid binding	NM_053113.2	Ribonuclease, RNase A family, 2A (liver, eosinophil-derived neurotoxin)	*Rnase2a*	−4.5	0.001
Sulfotransferase activity	NM_009286.2	Sulfotransferase family 2A, member 2	*Sult2a2*	−4.4	0.001
Response to oxidative stress	XM_001478443.6	Predicted NADH:ubiquinone oxidoreductase subunit B4B	*Ndufb4b*	−4.3	0.0000
Sulfation of steroids and bile acids	NM_001111296.2	Sulfotransferase family 2A, member 1	*Sult2a1*	−4.0	0.0000
Hydrogen peroxide catabolic process and oxygen transport	NM_001278161.1	Hemoglobin, beta adult major chain	*Hbb-b1*	−4.0	0.0000
NA	NA	Small nuclear ribonucleoprotein F	*Gm13092*	−4.0	0.0000
Ion transport	XM_006509537.4	Predicted solute carrier family 5 (sodium iodide symporter)	*Slc5a5*	−3.9	0.0002
Cell differentiation and protein transport	NM_001347632.2	Deleted in malignant brain tumors 1	*Dmbt1*	−3.9	0.0000
P450 pathway	NM_007813.2	Cytochrome P450, family 2, subfamily b, polypeptide 13	*Cyp2b13*	−3.7	0.0000
G protein-coupled receptor signaling pathway	NM_010999.3	Olfactory receptor 56p	*Olfr56*	−3.3	0.0000
Cation transport	NM_172583.3	Transmembrane protein 63c	*Tmem63c*	−3.2	0.0000
Regulation of GTPase activity	NM_011178.2	Proteinase 3	*Prtn3*	−3.2	0.0000
Ions and reactive oxygen species responses	NM_134066.3	Aldo-keto reductase family 1, member C18	*Akr1c18*	−3.0	0.0000
NA	NA	Circumsporozoite protein-like	*LOC108167857*	−2.7	0.0007
Cell adhesion and blood coagulation	NM_001001999.1	Glycoprotein Ib, beta polypeptide	*Gp1bb*	−2.7	0.0001
NA	NM_001013773.3	Neurexophilin and PC-esterase domain family, member 5	*Nxpe5*	−2.6	0.0005
Signaling pathway	NM_001101656.2	CD300 molecule like family member D4	*Cd300ld4*	−2.6	0.0000
Regulation of transcription	NG_065348.1	Coiled-coil-helix-coiled-coil-helix domain containing 2, pseudogene on chromosome 4	*Chchd2-ps*	−2.6	0.0000
Carbohydrate catabolism	NM_001042711.2	Amylase 2a5	*Amy2a5*	−2.5	0.0000
Cell-matrix adhesion	NM_080457.3	Mucin 4	*Muc4*	−2.3	0.0006
Transcription	NM_001346707	Predicted gene 3055	*Gm3055*	−2.3	0.001
G-protein coupled receptor signaling pathway	NM_013564.7	Insulin-like 3	*Insl3*	−2.2	0.0000
Immune response	NM_011280.2	Tripartite motif-containing 10	*Trim10*	−2.2	0.0001
Signaling pathway	NM_010014.3	Disabled 1	*Dab1*	−2.1	0.0000
Cell growth and differentiation	NM_010052.5	Delta like non-canonical Notch ligand 1	*Dlk1*	−2.1	0.0002
Regulation of cell adhesion	NM_001351947.1	Olfactomedin 4	*Olfm4*	−2.1	0.0000
Ion transport	NM_172469.3	Chloride intracellular channel 6	*Clic6*	−2.1	0.0000
Oxidoreductase activity	NM_021509.5	Monooxygenase, DBH-like 1	*Moxd1*	−2.0	0.0000
P450 pathway	NM_010000.2	Cytochrome P450, family 2, subfamily b, polypeptide 9	*Cyp2b9*	−2.0	0.0000
Ion binding	NM_009789.2	S100 calcium binding protein G	*S100g*	−2.0	0.0000
Immune response	NM_001013832.2	G protein-coupled receptor 31, D17Leh66b region	*Gpr31b*	−2.0	0.0003
Proteolysis	NM_010810.5	Matrix metallopeptidase 7	*Mmp7*	−2.0	0.0000
Oxidation process	NM_019545.4	Hydroxyacid oxidase 2	*Hao2*	−1.9	0.0000
Visual perception.	NM_021352.3	Crystallin, beta B3	*Crybb3*	−1.9	0.0000
Iron-binding	NM_008522.3	Lactotransferrin	*Ltf*	−1.9	0.0000
Glycolytic process	NM_001025388.2	Enolase 1B	*Eno1b*	−1.9	0.0000
Chromatin organization	NM_178187.4	H2A clustered histone 8	*Hist1h2ae*	−1.7	0.0000
Signaling pathway and hydrogen peroxide catabolism	NM_001362755.1	Dual oxidase 2	*Duox2*	−1.7	0.0000
mRNA splicing	NM_183024.1	Ribonucleoprotein, PTB-binding 2	*Raver2*	−1.7	0.0000
Cytoplasmic translation	NM_026517.3	Ribosomal protein L22 like 1	*Rpl22l1*	−1.7	0.00000
Keratinization	NM_009264.2	Small proline-rich protein 1A	*Sprr1a*	−1.6	0.0000
Carbohydrate and MHC class I protein binding	NM_133203.5	Killer cell lectin-like receptor, subfamily A, member 17	*Klra17*	−1.6	0.001
Modulation of synaptic transmission	NM_023716.2	Tubulin, beta 2B class IIB	*Tubb2b*	−1.6	0.0000
Ion transmembrane transport	NM_146017.3	Gamma-aminobutyric acid (GABA) A receptor, pi	*Gabrp*	−1.6	0.0002
G protein-coupled receptor activity	NM_001104614.1	Vomeronasal 2, receptor 3	*Vmn2r3*	−1.6	0.0000
Sulfotransferase activity	NM_001184981.2	Sulfotransferase family 2A, member 7	*Sult2a7*	−1.6	0.0000
Interferon response	NM_011579.3	T cell specific GTPase 1	*Tgtp1*	−1.5	0.0000
Transcription factor	NM_001033123.3	Predicted gene 14288	*Gm14288*	−1.5	0.0000
Binding monosaccharides	NM_001134644.1	Major urinary protein 13	*Mup13*	−1.5	0.0000
Regulation of gene expression, protein kinase B signaling	NM_001163011.1	Major urinary protein 1	*Mup1*	−1.5	0.0000

NA, not available.

**Table 3:** Changes in selected hepatic gene expressions of male *Apoe*-deficient mice receiving 10 mg/kg erythrodiol according to RT-qPCR assay.

Gene Symbol	Control (*n* = 14)	Erythrodiol (*n* = 15)	Fold Change	SL_2_R
*H4c17*	1.1 ± 0.5	1.0 ± 0.3	0.85	−0.23
*LOC100862456*	1.0 ± 0.3	1.1 ± 0.9	1.1	0.13
*Ccl19-ps2*	1.0 ± 0.2	0.7 ± 0.3 *	0.67	−0.58
*Ctrb1*	2.5 ± 3.4	100 ± 341	40	5.32
*Cyp2b10*	1.5 ± 1.4	2.3 ± 5.4 *	1.46	0.55
*Zfp969*	1.9 ± 2.0	2.5 ± 1.3	1.34	0.42
*Zfp965*	1.2 ± 0.6	1.5 ± 0.7	1.55	0.63
*Ttn*	1.3 ± 0.8	1.1 ± 0.7	0.90	−0.16
*Rbm14-rbm4*	1.0 ± 0.2	0.8 ± 0.3 *	0.77	−0.37
*Sec61g*	4.6 ± 9.4	0.1 ± 0.01 *	0.01	−6.54
*Rbm24*	1.9 ± 3.4	0.8 ± 0.7	0.44	−1.19
*Tmem81*	1.1 ± 0.5	0.6 ± 0.2 *	0.49	−1.02
*Rnase2a*	1.8 ± 2.2	1.4 ± 1.5	0.79	−0.34
*Sult2a2*	4.6 ± 13	2.1 ± 3.9	0.45	−1.15
*Ndufb4b*	1.1 ± 0.6	1.1 ± 0.5	0.97	−0.04
*Dmbt1*	1.4 ± 2.0	0.01 ± 0.01	0.01	−6.81
*Cyp2b13*	46 ± 105	6.2 ± 19	0.14	−2.89
*Prtn3*	7.4 ± 17	0.3 ± 0.2 *	0.04	−4.79
*Amy2a5*	1.1 ± 0.4	0.4 ± 0.1 *	0.41	−1.28
*Cyp2b9*	21 ± 31	1.0 ± 1.9 *	0.05	−4.36
*Mup1*	1.4 ± 0.8	0.6 ± 0.5 *	0.44	−1.19

Results are expressed as means and standard deviations normalized to the average of *Ppib* and *Tbp* as reference genes. Statistical analysis was carried out according to Mann–Whitney U-test and *, *p* < 0.05.

**Table 4:** Hepatic changes in selected gene expressions of female. *Apoe*-deficient mice receiving 10 mg/kg erythrodiol.

Gene Symbol	Control (*n* = 12)	Erythrodiol (*n* = 13)
*Cyp2b10*	1.6 ± 1.4	1.4 ± 1.5
*Dmbt1*	1.9 ± 2.8	6.5 ± 9.6
*Cyp2b13*	19 ± 18	17 ± 17
*Prtn3*	1.4 ± 1.4	1.5 ± 1.0
*Cyp2b9*	5.7 ± 4.1	5.2 ± 4.3

Results as arbitrary units according to RT-qPCR assay normalized to *Ppib* and *Tbp* are expressed as means and standard deviations. Statistical analysis was carried out according to Mann–Whitney U-test.

**Table 5:** Hepatic changes in selected gene expressions of male *Apoe*-deficient mice receiving different doses of erythrodiol.

Gene Symbol	Control (*n* = 17)	0.5 mg/kg Erythrodiol (*n* = 16)	1 mg/kg Erythrodiol (*n* = 17)	5 mg/kg Erythrodiol (*n* = 17)
*Cyp2b10*	1.2 ± 0.7	11.1 ± 41.3	1.6 ± 1.1	1.7 ± 1.8
*Dmbt1*	4.5 ± 15	2.5 ± 4.3	8.5 ± 27.0	1.7 ± 2.5
*Amy2a5*	1.0 ± 0.2	1.3 ± 0.2	1.1 ± 0.2	1.1 ± 0.4
*Prtn3*	1.9 ± 2.9	1.1 ± 1.2	3.2 ± 7.8 *	1.2 ± 1.0
*Cyp2b9*	1.6 ± 1.3	3.0 ± 2.6 *	2.2 ± 1.9	2.1 ± 1.7

Results are expressed as means and standard deviations according to RT-qPCR assay normalized to *Ppib* and *Tbp*. Statistical analysis was carried out according to One-way ANOVA and Mann–Whitney’s U-test for pair wise comparisons. *, *p* < 0.05 vs. control.

**Table 6:** Effect of 10 mg/kg erythrodiol on selected gene expressions in *Apoa1*-deficient mice according to sex.

Gene Symbol	Males		Females	
	Control (*n* = 14)	Erythrodiol (*n* = 15)	Control (*n* = 9)	Erythrodiol (*n* = 9)
*Cyp2b10*	30 ± 110	1.2 ± 1.8	7.2 ± 19	0.9 ± 0.9
*Dmbt1*	5.0 ± 10.8	7.3 ± 19	9.8 ± 21	87 ± 156
*Cyp2b13*	4.2 ± 6.3	4.5 ± 9.8	1.3 ± 0.9	1.9 ± 1.1
*Prtn3*	1.2 ± 0.8	1.1 ± 0.8	1.2 ± 0.9	5.3 ± 8.1
*Cyp2b9*	2.4 ± 3.8	1.6 ± 1.9	1.1 ± 0.5	1.2 ± 0.7

Results are expressed as means and standard deviations according to RT-qPCR assay normalized to *Ppib* and *Tbp*. Statistical analysis was carried out according to one-way ANOVA and Mann–Whitney’s U-test for pair-wise comparisons.

## Supplementary Materials

The following are available online at https://www.mdpi.com/1422-0067/21/19/7331/s1. Figure S1 and Table S1.

## References

[1] Keys A. (1995). Mediterranean diet and public health: Personal reflections. Am. J. Clin. Nutr..

[2] Trichopoulou A., Costacou T., Bamia C., Trichopoulos D. (2003). Adherence to a mediterranean diet and survival in a greek population. N. Engl. J. Med..

[3] Estruch R., Ros E., Salas-Salvado J., Covas M.I., Corella D., Aros F., Gomez-Gracia E., Ruiz-Gutierrez V., Fiol M., Lapetra J. (2018). Primary prevention of cardiovascular disease with a mediterranean diet supplemented with extra-virgin olive oil or nuts. N. Engl. J. Med..

[4] Martínez-González M.A., Gea A., Ruiz-Canela M. (2019). The mediterranean diet and cardiovascular health. Circ. Res..

[5] Foscolou A., Critselis E., Panagiotakos D. (2018). Olive oil consumption and human health: A narrative review. Maturitas.

[6] Lou-Bonafonte J.M., Arnal C., Navarro M.A., Osada J. (2012). Efficacy of bioactive compounds from extra virgin olive oil to modulate atherosclerosis development. Mol. Nutr. Food Res..

[7] Habib L., Jraij A., Khreich N., Charcosset C., Greige-Gerges H. (2015). Effect of erythrodiol, a natural pentacyclic triterpene from olive oil, on the lipid membrane properties. J. Membr. Biol..

[8] De la Puerta R., Martinez-Dominguez E., Ruiz-Gutierrez V. (2000). Effect of minor components of virgin olive oil on topical antiinflammatory assays. Zeitschrift für Naturforschung C.

[9] Acín S., Navarro M.A., Perona J.S., Surra J.C., Guillen N., Arnal C., Sarría A.J., Arbonés-Mainar J.M., Carnicer R., Ruiz-Gutiérrez V. (2007). Microarray analysis of hepatic genes differentially expressed in the presence of the unsaponifiable fraction of olive oil in apolipoprotein e-deficient mice. Br. J. Nutr..

[10] Juan M.E., Wenzel U., Daniel H., Planas J.M. (2008). Erythrodiol, a natural triterpenoid from olives, has antiproliferative and apoptotic activity in ht-29 human adenocarcinoma cells. Mol. Nutr. Food Res..

[11] Perona J.S., Arcemis C., Ruiz-Gutierrez V., Catala A. (2005). Effect of dietary high-oleic-acid oils that are rich in antioxidants on microsomal lipid peroxidation in rats. J. Agric. Food Chem..

[12] Abbass H.S., Ragab E.A., El-Salam A., Mohammed I., El-Hela A.A. (2015). Phytochemical and biological investigation of ficus mysorensis cultivated in egypt. J. Pharm. Chem. Biol. Sci..

[13] Abboud R., Charcosset C., Greige-Gerges H. (2016). Tetra- and penta-cyclic triterpenes interaction with lipid bilayer membrane: A structural comparative study. J. Membr. Biol..

[14] Marquez-Martin A., De La Puerta R., Fernandez-Arche A., Ruiz-Gutierrez V., Yaqoob P. (2006). Modulation of cytokine secretion by pentacyclic triterpenes from olive pomace oil in human mononuclear cells. Cytokine.

[15] Kontogianni V.G., Tsoumani M.E., Kellici T.F., Mavromoustakos T., Gerothanassis I.P., Tselepis A.D., Tzakos A.G. (2016). Deconvoluting the dual antiplatelet activity of a plant extract. J. Agric. Food Chem..

[16] Liu K., Qin Y.H., Yu J.Y., Ma H., Song X.L. (2016). 3-beta-epsilonrythrodiol isolated from conyza canadensis inhibits mkn45 human gastric cancer cell proliferation by inducing apoptosis, cell cycle arrest, DNA fragmentation, ros generation and reduces tumor weight and volume in mouse xenograft model. Oncol. Rep..

[17] Chen H.L., Lin K.W., Huang A.M., Tu H.Y., Wei B.L., Hour T.C., Yen M.H., Pu Y.S., Lin C.N. (2010). Terpenoids induce cell cycle arrest and apoptosis from the stems of celastrus kusanoi associated with reactive oxygen species. J. Agric. Food Chem..

[18] Nkengfack A.E., Azebaze A.G., Waffo A.K., Fomum Z.T., Meyer M., van Heerden F.R. (2001). Cytotoxic isoflavones from erythrina indica. Phytochemistry.

[19] Ebeling S., Naumann K., Pollok S., Wardecki T., Vidal Y.S.S., Nascimento J.M., Boerries M., Schmidt G., Brandner J.M., Merfort I. (2014). From a traditional medicinal plant to a rational drug: Understanding the clinically proven wound healing efficacy of birch bark extract. PLoS ONE.

[20] Xiaoli L., Naili W., Sau W.M., Chen A.S., Xinsheng Y. (2006). Four new isoflavonoids from the stem bark of erythrina variegata. Chem. Pharm. Bull..

[21] Allouche Y., Warleta F., Campos M., Sanchez-Quesada C., Uceda M., Beltran G., Gaforio J.J. (2011). Antioxidant, antiproliferative, and pro-apoptotic capacities of pentacyclic triterpenes found in the skin of olives on mcf-7 human breast cancer cells and their effects on DNA damage. J. Agric. Food Chem..

[22] Martin R., Ibeas E., Carvalho-Tavares J., Hernandez M., Ruiz-Gutierrez V., Nieto M.L. (2009). Natural triterpenic diols promote apoptosis in astrocytoma cells through ros-mediated mitochondrial depolarization and jnk activation. PLoS ONE.

[23] Allouche Y., Beltran G., Gaforio J.J., Uceda M., Mesa M.D. (2010). Antioxidant and antiatherogenic activities of pentacyclic triterpenic diols and acids. Food Chem. Toxicol..

[24] Martin R., Miana M., Jurado-Lopez R., Martinez-Martinez E., Gomez-Hurtado N., Delgado C., Bartolome M.V., San Roman J.A., Cordova C., Lahera V. (2012). Diol triterpenes block profibrotic effects of angiotensin ii and protect from cardiac hypertrophy. PLoS ONE.

[25] Martin R., Hernandez M., Cordova C., Nieto M.L. (2012). Natural triterpenes modulate immune-inflammatory markers of experimental autoimmune encephalomyelitis: Therapeutic implications for multiple sclerosis. Br. J. Pharm..

[26] Rajkumar A.P., Qvist P., Lazarus R., Lescai F., Ju J., Nyegaard M., Mors O., Borglum A.D., Li Q., Christensen J.H. (2015). Experimental validation of methods for differential gene expression analysis and sample pooling in rna-seq. BMC Genom..

[27] Peng X., Wood C.L., Blalock E.M., Chen K.C., Landfield P.W., Stromberg A.J. (2003). Statistical implications of pooling rna samples for microarray experiments. BMC Bioinform..

[28] Gabas-Rivera C., Martinez-Beamonte R., Rios J.L., Navarro M.A., Surra J.C., Arnal C., Rodriguez-Yoldi M.J., Osada J. (2013). Dietary oleanolic acid mediates circadian clock gene expression in liver independently of diet and animal model but requires apolipoprotein a1. J. Nutr. Biochem..

[29] Guillen N., Acin S., Surra J.C., Arnal C., Godino J., Garcia-Granados A., Muniesa P., Ruiz-Gutierrez V., Osada J. (2009). Apolipoprotein e determines the hepatic transcriptional profile of dietary maslinic acid in mice. J. Nutr. Biochem..

[30] Herrera-Marcos L.V., Sancho-Knapik S., Gabas-Rivera C., Barranquero C., Gascon S., Romanos E., Martinez-Beamonte R., Navarro M.A., Surra J.C., Arnal C. (2020). Pgc1a is responsible for the sex differences in hepatic cidec/fsp27beta mrna expression in hepatic steatosis of mice fed a western diet. Am. J. Physiol. Endocrinol. Metab..

[31] Zhao M., Zhao H., Lin L., Wang Y., Chen M., Wu B. (2020). Nuclear receptor co-repressor rip140 regulates diurnal expression of cytochrome p450 2b10 in mouse liver. Xenobiotica.

[32] Koga T., Yao P.L., Goudarzi M., Murray I.A., Balandaram G., Gonzalez F.J., Perdew G.H., Fornace A.J., Peters J.M. (2016). Regulation of cytochrome p450 2b10 (cyp2b10) expression in liver by peroxisome proliferator-activated receptor-beta/delta modulation of sp1 promoter occupancy. J. Biol. Chem..

[33] Gabás-Rivera C., Jurado-Ruiz E., Sánchez-Ortiz A., Romanos E., Martínez-Beamonte R., Navarro M.A., Surra J.C., Arnal C., Rodríguez-Yoldi M.J., Cristina Andrés-Lacueva C. (2020). Dietary squalene induces cytochromes cyp2b10 and cyp2c55 independently of sex, dose and diet in several mouse models. Mol. Nutr. Food Res..

[34] Heintz M.M., Kumar R., Rutledge M.M., Baldwin W.S. (2019). Cyp2b-null male mice are susceptible to diet-induced obesity and perturbations in lipid homeostasis. J. Nutr. Biochem..

[35] Mirea A.M., Stienstra R., Kanneganti T.D., Tack C.J., Chavakis T., Toonen E.J.M., Joosten L.A.B. (2020). Mice deficient in the il-1beta activation genes prtn3, elane, and casp1 are protected against the development of obesity-induced nafld. Inflammation.

[36] Hu D., Ansari D., Zhou Q., Sasor A., Said Hilmersson K., Andersson R. (2019). Low p4ha2 and high prtn3 expression predicts poor survival in patients with pancreatic cancer. Scand. J. Gastroenterol..

[37] Zhou Y., Jiang L., Rui L. (2009). Identification of mup1 as a regulator for glucose and lipid metabolism in mice. J. Biol. Chem..

[38] Fan Y., Fang X., Tajima A., Geng X., Ranganathan S., Dong H., Trucco M., Sperling M.A. (2014). Evolution of hepatic steatosis to fibrosis and adenoma formation in liver-specific growth hormone receptor knockout mice. Front. Endocrinol..

[39] Liu B., Liu J., Liao Y., Jin C., Zhang Z., Zhao J., Liu K., Huang H., Cao H., Cheng Q. (2019). Identification of sec61g as a novel prognostic marker for predicting survival and response to therapies in patients with glioblastoma. Med. Sci. Monit..

[40] Chen J., Qian Z., Li F., Li J., Lu Y. (2017). Integrative analysis of microarray data to reveal regulation patterns in the pathogenesis of hepatocellular carcinoma. Gut Liver.

[41] Sultana N., Rahman M., Myti S., Islam J., Mustafa M.G., Nag K. (2019). A novel knowledge-derived data potentizing method revealed unique liver cancer-associated genetic variants. Hum. Genom..

[42] Mollenhauer J., Wiemann S., Scheurlen W., Korn B., Hayashi Y., Wilgenbus K.K., Poustka A. (1997). Dmbt1, a new member of the srcr superfamily, on chromosome 10q25.3–26.1 is deleted in malignant brain tumours. Nat. Genet..

[43] Kwekel J.C., Desai V.G., Moland C.L., Branham W.S., Fuscoe J.C. (2010). Age and sex dependent changes in liver gene expression during the life cycle of the rat. BMC Genom..

[44] Xie X., Miao L., Yao J., Feng C., Li C., Gao M., Liu M., Gong L., Wang Y., Qi X. (2013). Role of multiple micrornas in the sexually dimorphic expression of cyp2b9 in mouse liver. Drug Metab. Dispos..

[45] Kumar R., Mota L.C., Litoff E.J., Rooney J.P., Boswell W.T., Courter E., Henderson C.M., Hernandez J.P., Corton J.C., Moore D.D. (2017). Compensatory changes in cyp expression in three different toxicology mouse models: Car-null, cyp3a-null, and cyp2b9/10/13-null mice. PLoS ONE.

[46] Jarukamjorn K., Sakuma T., Nemoto N. (2000). Discriminating activation of cyp2b9 expression in male c57bl/6 mouse liver by beta-estradiol. Biochem. Biophys. Res. Commun..

[47] Sato Y., Kaneko Y., Cho T., Goto K., Otsuka T., Yamamoto S., Goto S., Maruyama H., Narita I. (2017). Prolactin upregulates female-predominant p450 gene expressions and downregulates male-predominant gene expressions in mouse liver. Drug Metab. Dispos..

[48] Sanchez-Quesada C., Lopez-Biedma A., Warleta F., Campos M., Beltran G., Gaforio J.J. (2013). Bioactive properties of the main triterpenes found in olives, virgin olive oil, and leaves of olea europaea. J. Agric. Food Chem..

[49] Arbonés-Mainar J.M., Navarro M.A., Acín S., Guzmán M.A., Arnal C., Surra J.C., Carnicer R., Roche H.M., Osada J. (2006). Trans-10, cis-12- and cis-9, trans-11-conjugated linoleic acid isomers selectively modify hdl-apolipoprotein composition in apolipoprotein e knockout mice. J. Nutr..

[50] Gimenez E., Juan M.E., Calvo-Melia S., Planas J.M. (2017). A sensitive liquid chromatography-mass spectrometry method for the simultaneous determination in plasma of pentacyclic triterpenes of *Olea europaea* L.. Food Chem..

[51] Acin S., Navarro M.A., Perona J.S., Arbones-Mainar J.M., Surra J.C., Guzman M.A., Carnicer R., Arnal C., Orman I., Segovia J.C. (2007). Olive oil preparation determines the atherosclerotic protection in apolipoprotein e knockout mice. J. Nutr. Biochem..

[52] Surra J.C., Guillen N., Arbones-Mainar J.M., Barranquero C., Navarro M.A., Arnal C., Orman I., Segovia J.C., Osada J. (2010). Sex as a profound modifier of atherosclerotic lesion development in apolipoprotein e-deficient mice with different genetic backgrounds. J. Atheroscler. Thromb..

[53] Li H., Reddick R.L., Maeda N. (1993). Lack of apoa-i is not associated with increased susceptibility to atherosclerosis in mice. Arter. Thromb..

[54] Gabas-Rivera C., Barranquero C., Martinez-Beamonte R., Navarro M.A., Surra J.C., Osada J. (2014). Dietary squalene increases high density lipoprotein-cholesterol and paraoxonase 1 and decreases oxidative stress in mice. PLoS ONE.

[55] Carnicer R., Navarro M.A., Arbones-Mainar J.M., Arnal C., Surra J.C., Acin S., Sarria A., Blanco-Vaca F., Maeda N., Osada J. (2007). Genetically based hypertension generated through interaction of mild hypoalphalipoproteinemia and mild hyperhomocysteinemia. J. Hypertens..

[56] Guillen N., Acin S., Navarro M.A., Perona J.S., Arbones-Mainar J.M., Arnal C., Sarria A.J., Surra J.C., Carnicer R., Orman I. (2008). Squalene in a sex-dependent manner modulates atherosclerotic lesion which correlates with hepatic fat content in apoe-knockout male mice. Atherosclerosis.

[57] Pertea M., Pertea G.M., Antonescu C.M., Chang T.-C., Mendell J.T., Salzberg S.L. (2015). Stringtie enables improved reconstruction of a transcriptome from rna-seq reads. Nat. Biotechnol..

[58] Trapnell C., Roberts A., Goff L., Pertea G., Kim D., Kelley D.R., Pimentel H., Salzberg S.L., Rinn J.L., Pachter L. (2012). Differential gene and transcript expression analysis of rna-seq experiments with tophat and cufflinks. Nat. Protoc..

[59] Kong L., Zhang Y., Ye Z.-Q., Liu X.-Q., Zhao S.-Q., Wei L., Gao G. (2007). Cpc: Assess the protein-coding potential of transcripts using sequence features and support vector machine. Nucleic Acids Res..

[60] Shen S., Park J.W., Lu Z.-X., Lin L., Henry M.D., Wu Y.N., Zhou Q., Xing Y. (2014). Rmats: Robust and flexible detection of differential alternative splicing from replicate rna-seq data. Proc. Natl. Acad. Sci. USA.

[61] Langmead B., Salzberg S.L. (2012). Fast gapped-read alignment with bowtie 2. Nat. Methods.

[62] Li B., Dewey C.N. (2011). Rsem: Accurate transcript quantification from rna-seq data with or without a reference genome. BMC Bioinform..

[63] Untergasser A., Cutcutache I., Koressaar T., Ye J., Faircloth B.C., Remm M., Rozen S.G. (2012). Primer3—New capabilities and interfaces. Nucleic Acids Res..

