# Supplementary material for: Dietary Erythrodiol Modifies Hepatic Transcriptome in Mice in a Sex and Dose-Dependent Way
Source: Int J Mol Sci. 2020 Oct 4;21(19):7331. doi: 10.3390/ijms21197331 (PMC7582860; doi:10.3390/ijms21197331)

Supplementary Table 1. Characteristics of primers used in RT-qPCR according to MIQE guidelines.

Gene Symbol	Accession		Sequence	Amplicon Length	Exons	[Primer]	Efficiency
H4c17	NM_001195421.1	Sense<br>Antisense	CAAAGTGCTGCGCGATAACA<br>AGATGCGCTTCACTCCTCC	83	1	0.2 μM	94%
LOC100862456	XM_006537451.3	Sense<br>Antisense	TTGTGGAGAGTGTCACACCG<br>TCGAGCATCAAAAACCACCA	121	1	0.05 μM	92%
Ccl19-ps2	XM_006536385.3	Sense<br>Antisense	GTAGGAGCCTCGACCTCTCA<br>GGAAGGTCCAGAGAACCAGC	142	1	0.2 μM	94%
Ctrb1	NM_025583.2	Sense<br>Antisense	CACCACCATGGCATTCCTTT<br>GCATCCTCTCCGTTGACGAT	126	1/2	0.075 μM	94%
Cyp2b10	NM_009999.4	Sense<br>Antisense	ATGTTTAGTGGAGGAACTGCGG<br>ATATTGGCCGTGATGCACTG	81	3/4	0.1 μM	103%
Zfp969	XM_017319408.2	Sense<br>Antisense	ATGAAAGAGAAACCCTATGAATGC<br>ACACCCCTGTATCCTCTTGA	112	1	0.2 μM	96%
Zfp965	NM_001242944.1	Sense<br>Antisense	CTTCACTCAGGAAGAGTGGGC<br>TGCCTTTGACTACGACTCTGA	243	2/4	0.1 μM	95%
Ttn	NM_011652.3	Sense<br>Antisense	GCCACTGCTGTGGTAGAAGT<br>CTTAGACCCGCCATCGTCTC	129	245/246	0.2 μM	98%
Rbm14-rbm4	NM_001290127.1	Sense<br>Antisense	AATGTCGATGGGGCGGATAC<br>CAGCTCATGACCGTGCCATA	74	1	0.2 μM	106%
Sec61g	XM_003085302.1	Sense<br>Antisense	TTGTTGAGCCAAGTCAGCGG<br>TGATAGCAAATCCTATCGTTGTGG	120	1/2	0.05 μM	105%
Rbm24	NM_001081425.1	Sense<br>Antisense	TGCAGCCAGGTTTTGCCTTT<br>ATGTACGGTGTGGTGGAAGCC	166	2/4	0.2 μM	95%
Tmem81	NM_029025.3	Sense<br>Antisense	TGGCCTTGGCTACAAGGAAG<br>AATTGGCTCGGAAGGGTCTG	266	2	0.1 μM	93%
Rnase2a	NM_053113.2	Sense<br>Antisense	GAGCCTATCCCCGATGTGATG<br>ATGACTGGCCGGAGTTGTG	218	2	0.1 μM	93%

Sult2a2	NM_009286.2	Sense<br>Antisense	ACCTCCCATCTTCCCATCCA<br>TCATGAGATAGATCGCCTTGG	73	2/3	0.1 μM	95%
Ndufb4b	XM_001478443.6	Sense<br>Antisense	TGAAAATCCCGGGGAGTCAA<br>CGGAGACACGTCATACTCGG	102	1	0.2 μM	94%
Dmbt1	NM_001347632.2	Sense<br>Antisense	CACCACCAATCTCCTTTGTCAG<br>GTCTCGTTGTCAGCCTGTTTGA	207	44/46	0.1 μM	99%
Cyp2b13	NM_007813.2	Sense<br>Antisense	CTCATGCTGAGTCACTTCCCTCTT<br>ACAGACCACAGAGTGTGAAGTTGG	52	9	0.1 μM	91%
Prtn3	NM_011178.2	Sense<br>Antisense	CAATTACAACCCCGAGGAGAAC<br>TTTAGCTGGAGGAGAAGCACGT	51	3/4	0.1 μM	95%
Amy2a5	NM_001042711.2	Sense<br>Antisense	TGCTTTCCCTCATTGGGTTC<br>AATATCAACCCAGCGCCACT	101	1	0.1 μM	93%
Cyp2b9	NM_010000.2	Sense<br>Antisense	ACCAGATCTACTTCTTAGCCCGCT<br>GAGAACAACAGTAGAAGGAAGGGTG	151	9	0.1 μM	104%
Mup1	NM_001163011.1	Sense<br>Antisense	TAAGAACAAGCAAAGGGGCTGG<br>TCAGTCCCAAACACAGCAGCA	127	1	0.1 μM	97%
Apoa1	NM_009692.4	Sense<br>Antisense	GCTGAACCTGAATCTCCTGGAA<br>ACTAACGGTTGAACCCAGAGTGTC	52	3/4	0.1 μM	-
Apoe	NM_009696.4	Sense<br>Antisense	CTTGTTTCGGAAGGAGCTGACT<br>AGGCATCCTGTCAGCAATGTG	92	1/3	0.1 μM	-
Tbp	NM_013684.3	Sense<br>Antisense	GTGAGTTGCTTGCTCTGTGC<br>GCTGCGTTTTTGTGCAGAGT	359	8	0.2 μM	104%
Pipb	NM_011149	Sense<br>Antisense	GGAGATGGCACAGGAGGAA<br>GTAGTGCTTCAGCTTGAAGTTCTCAT	72	3/4	0.1 μM	103%

Supplementary Figure 1. PCR products obtained from hepatic RNA by RT-PCR. *Apoa1*-deficient mice do not express *Apoa1* and *Apoe*-deficient mice do not express *Apoe*. Lane 1, *Apoa1*-deficient mice; lane 2, *Apoe*-deficient mice and lane 3, negative control, no cDNA input. M, DNA ladder. PCR products were run into a 2% agarose gel and stained with ethidium bromide.

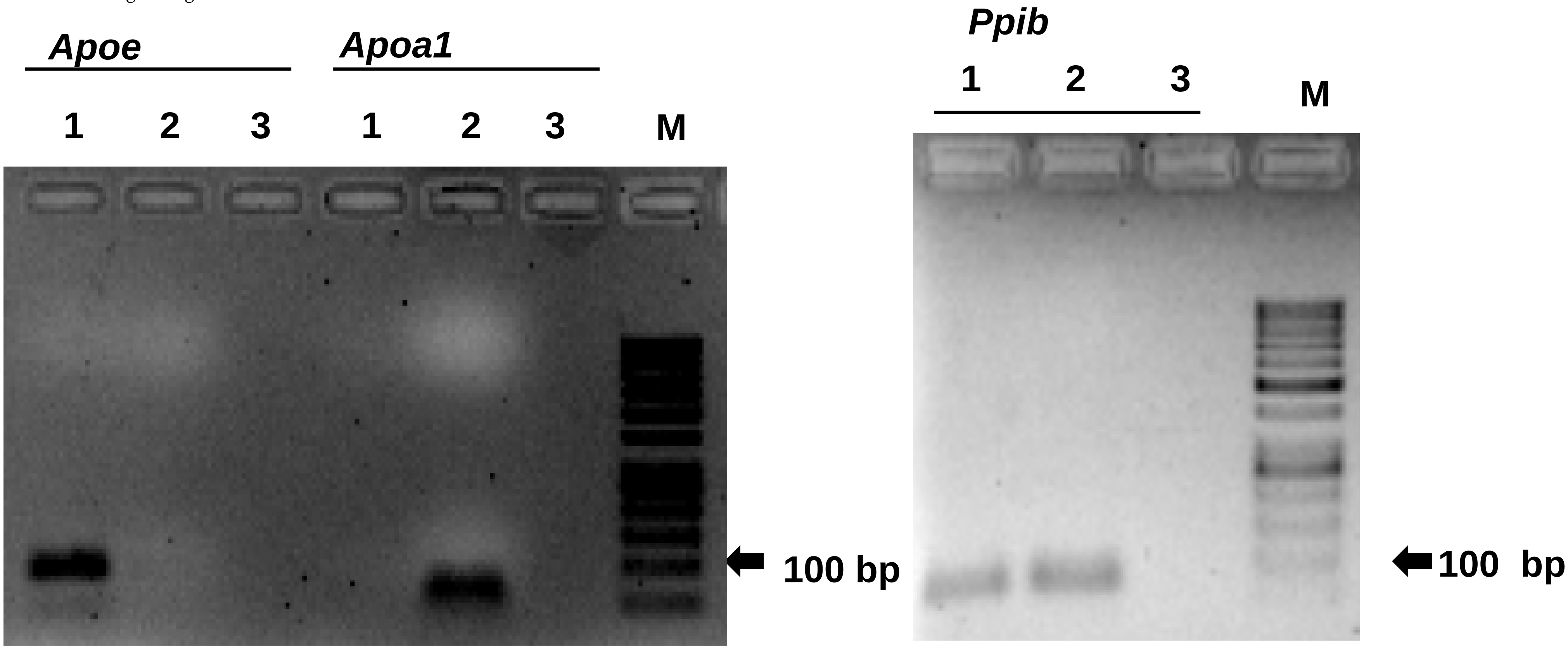

Supplement: Supplementary file 1 [file ijms-21-07331-s001.pdf]
